# Successful superselective embolization with *n*-butyl cyanoacrylate for hemorrhage from superficial femoral artery branches following blunt trauma: Three case reports

**DOI:** 10.1097/MD.0000000000020467

**Published:** 2020-07-02

**Authors:** Chang Hoon Oh, Yook Kim, Jung Hwan Lee, Hong Rye Kim, Seung Je Go

**Affiliations:** a Department of Radiology; b Department of Neurosurgery, Chungbuk National University Hospital; c Department of Surgery, Trauma Center, Eulji University Hospital, Cheongju, Republic of Korea.

**Keywords:** blunt arterial injury, embolization, *n*-butyl cyanoacrylate, superficial femoral artery

## Abstract

**Introduction:**

In blunt traumatic superficial femoral arterial (SFA) injuries, hemorrhage from the branches without injury to the main artery is rare, but can lead to serious complications, such as compartment syndrome affecting the clinical outcomes. Although open surgical repair has been the standard approach to peripheral vascular injuries, endovascular treatment has become more refined and is now an alternative to open surgery, which potentially involves lower morbidity and mortality rates. However, management of arterial injuries, especially when they involve simple bleeding from small branches of the main artery, can be challenging, and the best treatment options for such injuries remains controversial.

**Patient concerns:**

Three cases suffered blunt trauma that resulted in hemorrhage from branches of the SFA.

**Diagnosis:**

All patients underwent selective angiography, which demonstrated active extravasation from branches of the SFA.

**Interventions:**

All patients were treated using embolization with *n*-butyl cyanoacrylate (NBCA).

**Outcomes:**

A post-embolization angiography demonstrated successful hemostasis, with no complications.

**Conclusion:**

Superselective catheterization using a coaxial technique with a 5-F curved catheter and the smallest caliber microcatheter, and using a permanent liquid embolic agent, such as NBCA, increases the success rate of embolization for cases of hemorrhage from SFA branches.

## 1 Introduction

The superficial femoral artery (SFA) is the most common site for lower extremity arterial injuries, accounting for 37.2% of such injuries.^[1]^ Among these, hemorrhage from branches of the SFA, without accompanying injury to the main artery, after bunt trauma is extremely rare, and is frequently obscured by concomitant multiple injuries, but can potentially lead to fatal complications, such as compartment syndrome, which affect the clinical outcome.^[2,3]^

Endovascular repair has been widely employed in the management of traumatic arterial injuries, and with advances in technology, it is now associated with better in-hospital mortality and lower rates of sepsis than open surgery.^[4]^ A trauma lesion of the main artery in the lower limb can be treated with placement of a bare metal stent or a stent graft, whereas distal branch bleeding can be embolized with coils, gelfoam particles, liquid embolic agents (such as *n*-butyl cyanoacrylate; NBCA), or a combination of these approaches.^[5]^ However, embolization for bleeding from small branches of the SFA is technically more challenging and sometimes even impossible, due to the unfavorable vascular anatomy or small caliber of the vessels, and subsequently the best treatment option for such injuries remains controversial.

There is little evidence about the efficacy and safety of endovascular methods for hemorrhage from branches of the SFA, and there are a few reports where embolization failed, necessitating open surgery.^[2,6]^ Herein, we report 3 cases of hemorrhage from branches of the SFA after blunt trauma, all of which were successfully treated with embolization by NBCA using a coaxial technique with a 5-F curved catheter and the smallest caliber microcatheter.

The 2 survivors have provided informed consent for publication of the case. Meanwhile, we could not obtain consent from the deceased patient, but the Institutional Review Board (IRB) of the Chungbuk National University Hospital in Cheongju, Republic of Korea considered exempt of approval for publication of the case.

## 2 Case presentation

### 2.1 Case 1

**Figure 1:**
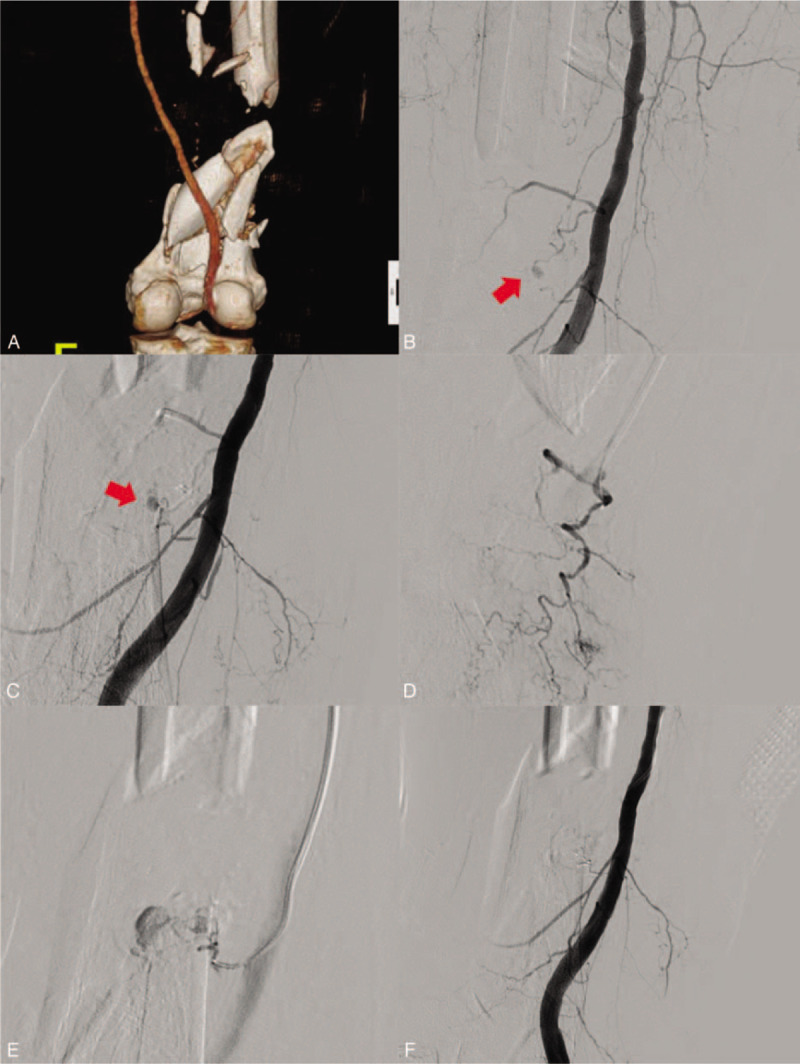
A 74-year-old man was admitted to our hospital after a car accident. (A) Initial lower extremity computed tomography (CT) revealed a comminuted fracture of the right distal femur. (B and C) Two pseudoaneurysms from a muscular branch (red arrow) and descending genicular branch (red arrow) were identified on right superficial femoral arterial (SFA) angiography. (D and E) Selective catheterization of the orifice of the bleeding branches was performed directly with a 5-F C2 catheter, and then a 1.7-F microcatheter was introduced into the proximal portion of the bleeding branch using a coaxial technique. Two bleeding branches were embolized with 1:2 mixtures of *n*-butyl cyanoacrylate (NBCA) and iodized oil. (F) A post-embolization angiography demonstrated successful hemostasis, with no complications.

A 74-year-old man was admitted to our hospital after a car accident, with multiple traumas that included a comminuted fracture of the right distal femur (Fig. 1A). Active bleeding in the right distal femur was revealed by computed tomography (CT) scan and subsequently, angiography was attempted. The right SFA was catheterized via left femoral artery access. Two pseudoaneurysms from the muscular branch (Fig. 1B) and descending genicular branch (Fig. 1C) were identified on right SFA angiography. Selective catheterization of the orifice of the bleeding branches was performed directly with a 5-F C2 catheter (Cook, Bloomington, IN), and then a 1.7-F microcatheter (ASHAI INTECC, Aichi, Japan) was introduced into the proximal portion of the bleeding branch, using the coaxial technique (Fig. 1D and E). Thereafter, the 2 bleeding branches were embolized with 1:2 mixtures of NBCA and iodized oil. A post-embolization angiography demonstrated successful hemostasis, with no complications (Fig. 1F). Three days after the embolization, the patient underwent an open reduction and internal fixation of the right distal femur. The patient recovered well and was discharged 12 weeks later. No complications were noted at the 8-month follow-up assessment.

### 2.2 Case 2

**Figure 2:**
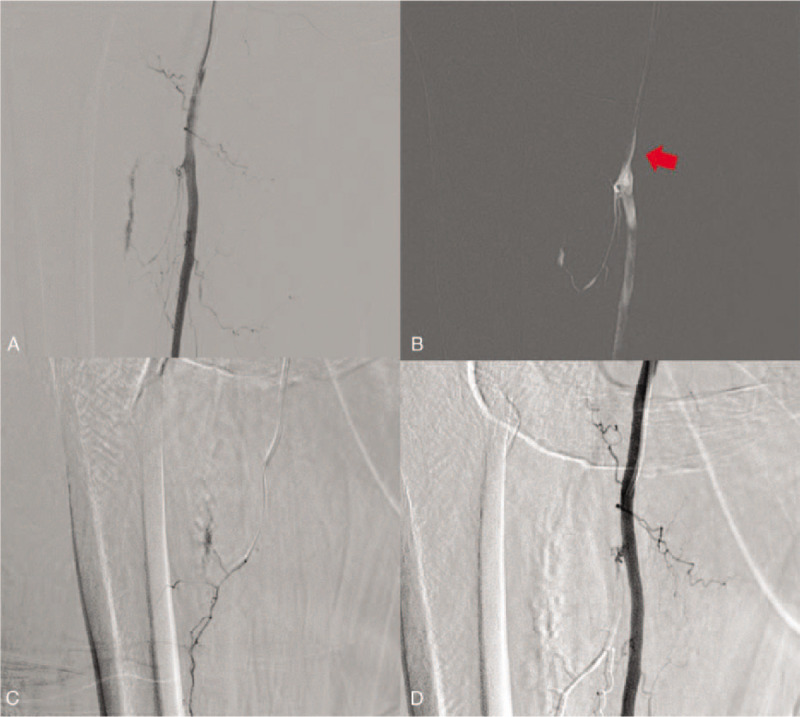
A 69-year-old woman presented to our hospital with multiple traumas after being hit by a car. (A) Selective angiography demonstrated active extravasation from a muscular branch of the right superficial femoral artery (SFA). (B) Selective catheterization of the orifice of the bleeding branches was performed directly with a 5-F C2 catheter (red arrow), and then (C) a 1.7-F microcatheter was introduced into the proximal portion of the bleeding branch using the coaxial technique. (D) Embolization was attempted with a 1:2 mixture of NBCA and iodized oil and final angiography demonstrated successful hemostasis. NBCA = *n*-butyl cyanoacrylate.

A 69-year-old woman presented to our hospital with multiple traumas after being hit by a car. A tense, tender swelling had developed in the right thigh with systolic blood pressure dropping to 70 mmHg. Therefore, a vascular injury caused by blunt trauma was strongly suspected. The patient was referred directly for emergency angiography without any imaging evaluation. The right SFA was catheterized via left femoral artery access and selective angiography demonstrated active extravasation from the muscular branch of the right SFA (Fig. 2A). Selective catheterization of the orifice of the bleeding branches was performed directly with a 5-F C2 catheter (Cook) (Fig. 2B), and then a 1.7-F microcatheter (ASHAI INTECC) was introduced into the proximal portion of the bleeding branch, using the coaxial technique (Fig. 2C). Next, embolization was attempted with a 1:2 mixture of NBCA and iodized oil. Final angiography demonstrated successful elimination of the active bleeding (Fig. 2D). There was no evidence of complications and the patient's vital signs improved after embolization. The patient recovered well and was discharged 8 weeks later.

### 2.3 Case 3

**Figure 3:**
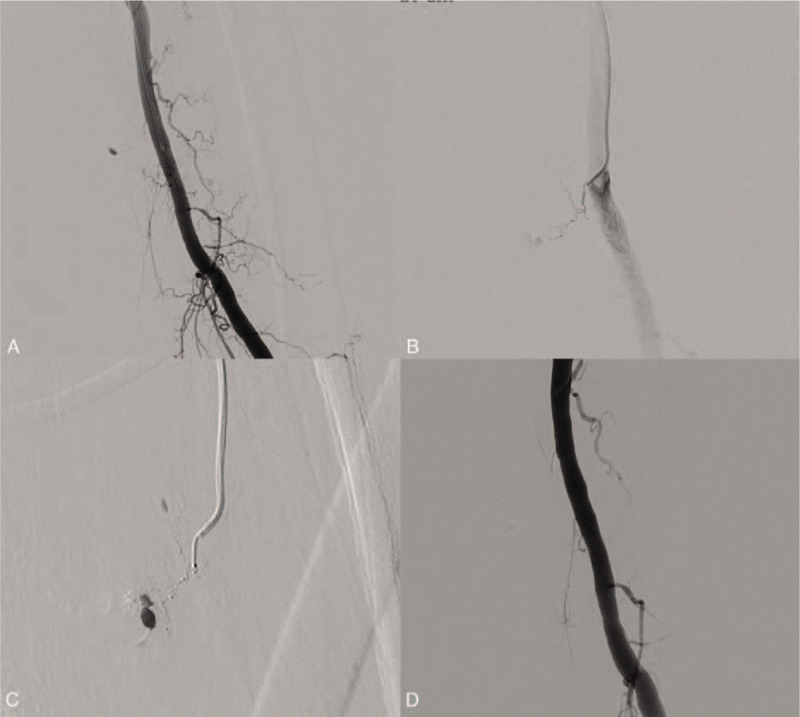
A 73-year-old man was admitted to the trauma center after being hit by a car, with multiple traumas. (A) Angiography of the left superficial femoral artery (SFA) was performed and revealed active extravasation from a muscular branch of the left SFA. (B) Selective catheterization of the orifice of the bleeding branch was performed directly with a 5-F C2 catheter and then (C) a 1.7-F microcatheter was introduced into the proximal portion of the bleeding branch using the coaxial technique. (D) Embolization was performed with a 1:2 mixture of NBCA and iodized oil. Contrast medium injection then demonstrated the disappearance of active bleeding. NBCA = *n*-butyl cyanoacrylate.

A 73-year-old man was admitted to the trauma center after being hit by a car, with multiple traumas. The patient was hemodynamically unstable and complained of severe pain in the left leg. CT scan revealed active bleeding from the second portion of duodenum and the patient underwent open surgery on admission. That evening, acute swelling and worsening pain developed in the left thigh; therefore, a vascular injury was strongly suspected. We proceeded to conduct angiography and right femoral arterial access was achieved with a 5-F sheath. Angiography of the left SFA was performed and revealed active extravasation from a muscular branch of the left SFA (Fig. 3A). Selective catheterization of the orifice of the bleeding branch was performed directly with a 5-F C2 catheter (Cook), and then 1.7-F microcatheter (ASHAI INTECC) was introduced into the proximal portion of the bleeding branch using the coaxial technique. Then, embolization was performed with a 1:2 mixture of NBCA and iodized oil (Lipiodol), followed by a contrast medium injection that demonstrated the disappearance of active bleeding. There was no evidence of procedure-related complications. However, 5 days after embolization, the patient died due to prolonged hypovolemic-related multiple organ failure, even though the bleeding in the thigh was controlled by embolization.

## 3 Discussion

Traumatic vascular injuries involving the extremities are rare, and in fact, only 26% of extremity traumas involve vascular lesions; among these, hemorrhage from small branches of SFA after bunt trauma is extremely rare.^[1,7]^ Historically, open repair has been the mainstay of surgical management for arterial vascular trauma.^[8,9]^ However, surgery usually necessitates general anesthesia and it may be challenging for surgeons to work in a field that is already distorted by injury, especially if the affected vessel is located deep in an obese patient.^[10]^ Endovascular repair yields a shorter hospital stay and requires a shorter period of inactivity than surgery.^[5]^ With improvements in medical technology and evolution of less invasive methods, transcatheter arterial embolization has been widely used in approaches for dealing with lower extremity arterial injury, as an alternative to open surgery.^[4,5,11]^ Nevertheless, the efficacy of embolization in hemorrhage from branches of the SFA has infrequently been reported; these reports involved a few patients where embolization has failed or could not be catheterized, and required open surgery.^[2,6]^

Although the injury pattern to the vessels has been suggested to play a role in determining the type of repair employed, regardless of the injury mechanism, the best treatment options for simple arterial bleeding from small branches remain controversial. Covered stents are attractive options for the management of SFA hemorrhages, particularly in areas that are difficult to expose surgically.^[5,11]^ However, covered stents could have disadvantages for younger patients with a long life expectancy, because the long-term consequences of implanting covered stents are not known.^[12]^ Conservative treatment could also be considered in cases of simple arterial bleeding from small branches, especially when patients are hemodynamically stable on admission. However, a conservative approach may result in complications, such as continuous bleeding and compression of the surrounding structures by the expanding hematoma^[6,7]^ and acute compartment syndrome of the thigh may result in blunt trauma patients.^[3]^ In 1 case report of conservative treatment of a patient with SFA branch bleeding, the patient eventually developed deep vein thrombosis and subsequent pulmonary embolism due to venous compression from the worsened hematoma.^[6]^ Therefore, embolization should be considered rather than conservative treatment, if it is technically possible, and it is not associated with a risk of ischemia distal to the lesion.

With advances in equipment and the introduction of new catheters and microcatheters, the frequency of unsuccessful catheterization has decreased.^[4,8,11,12]^ A selective technique is essential for avoiding technical failure or procedure-related complications. Therefore, we recommend that the following points be considered when embolization is performed in patients with hemorrhage from branches of the SFA. First, when selecting bleeding branches, a 5-F curved catheter, such as the Cobra II catheter, should be used. It is difficult to select the bleeding branches of the SFA directly with the microcatheter because the tip of the microcatheter is difficult to move in the desired direction, especially in wide diameter arteries such as the SFA. On the other hand, with a curved catheter, we can control the direction of the catheter tip, and consequently, it can be located as close to the bleeding branches as possible, or the orifice of bleeding branches can be selected directly, as shown in our cases (Figs. 1D, E, 2B, and 3B). It also helps to obtain a stable microcatheter position, to avoid catheter “kick-out” when introducing the microcatheter into the bleeding branches, and to prevent nontargeted embolization. Second, the smallest caliber microcatheter should be used, and it should be advanced as far as possible to the distal portion of the bleeding branches. In this way, a sufficient and complete embolic effect can be obtained while reducing the possibility of backflow of embolic material into the main artery, which could cause ischemic complications. In our cases, a 1.7-F microcatheter, the smallest caliber available in Korea, was introduced into the bleeding branches and rebleeding was not noted in any cases after embolization. Of course, the use of a small caliber may limit the use of effective embolic materials, such as microcoils, but liquid embolic agents, such as NBCA, can compensate for this, as shown in our cases. Finally, when performing embolization, NBCA should be considered as an embolic agent. To the best of our knowledge, no previous reports have described the use of NBCA as an embolic agent for controlling bleeding from branches of the SFA caused by blunt trauma. NBCA has the advantage of being rapidly delivered to the bleeding sites and has been used effectively as an embolic agent for penetrating arterial injury in the lower limbs.^[11]^ Other potential advantages of NBCA include its capacity to obliterate small distal vessels, and its effective occlusion of bleeding sites that have complex anatomical structures. However, the danger of damaging a limb's circulation due to reflux of the agent should be considered. To prevent this complication, an appropriate volume, speed of injection, and adjustable ratio of iodized oil to NBCA mixture should be used. In our cases, we used 1:2 mixtures of NBCA and iodized oil and 0.2 to 0.4 mL of the mixture, which was carefully injected under fluoroscopic monitoring. To avoid adhesion of the catheter tip to the vessel wall, the microcatheter was quickly removed after injection. Fortunately, there was no inadvertent embolization, which is associated with a risk of ischemia distal to the lesion (Table 1).

**Table 1:**
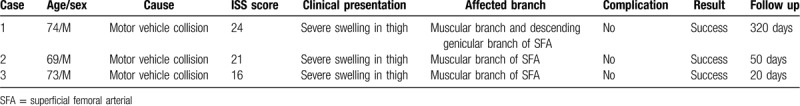
Clinical characteristics and outcome in 3 patients with hemorrhage from branches of superficial femoral artery after blunt trauma.

## 4 Conclusion

In conclusion, arterial embolization can be a feasible, safe, and effective option for treating traumatic vascular injuries involving the extremities, allowing for timely and minimally invasive management. Hemorrhage from branches of the SFA after blunt trauma is extremely rare, but can potentially lead to fatal complications, such as compartment syndrome. Embolization could be used for managing SFA branch bleeding if the vessels can be safely sacrificed without any anticipated ischemic sequelae. Therefore, performing directly selected catheterization of the orifice of the bleeding branches with a 5-F curved catheter, and then introducing a small caliber microcatheter using the coaxial technique, and using a permanent liquid embolic agent, such as NBCA, may increase the success rate of embolization.

## Author contributions

XXX
